# Hypothesis: Pentoxifylline is a potential cytokine modulator therapeutic in COVID‐19 patients

**DOI:** 10.1002/prp2.631

**Published:** 2020-07-26

**Authors:** Bruce M. Hendry, Nina Stafford, Ahran D. Arnold, Arvind Sangwaiya, Vijay Manglam, Stuart D. Rosen, Jayantha Arnold

**Affiliations:** ^1^ Renal Sciences Department of Inflammation Biology King’s College London London UK; ^2^ Department of Gastroenterology Ealing Hospital London North West University Healthcare NHS Trust Southall UK; ^3^ National Heart and Lung Institute Imperial College London London UK

**Keywords:** COVID‐19, cytokine, immunomodulation, pentoxifylline, pneumonia, SARS‐CoV‐2, viral

## Abstract

We propose a new hypothesis that the established drug pentoxifylline deserves attention as a potential repurposed therapeutic for COVID‐19. Pentoxifylline is an immunomodulator with anti‐inflammatory properties. It is a nonselective phosphodiesterase inhibitor and through Adenosine A2A Receptor‐mediated pathways reduces tumor necrosis factor alpha, interleukin 1, interleukin 6, and interferon gamma and may act to reduce tissue damage during the cytokine storm host response to SARS‐CoV‐2 infection. This agent has been used clinically for many years and has a favorable profile of safety and tolerability. Pre‐clinical data support pentoxifylline as effective in cytokine‐driven lung damage. Clinical studies of pentoxifylline in radiation and cytokine‐induced lung damage in humans are positive and consistent with anti‐inflammatory efficacy. Pentoxifylline is a readily available, off‐patent and inexpensive drug, suitable for large‐scale use including in resource‐limited countries. Current trials of therapeutics are largely focused on the inhibition of viral processes. We advocate urgent randomized trials of pentoxifylline for COVID‐19 as a complementary approach to target the host responses.

**Abbreviations**
ACE: angiotensin‐converting enzyme
AMP: adenosine monophosphate
ARDS: acute respiratory distress syndrome
AT1: Type 1 Angiotensin II receptor
CRP: C‐reactive protein
ICAM1: intercellular adhesion molecule 1
IFN gamma: interferon gamma
IkB: Inhibitory factor kappa B
IL: interleukin
IMP: inosine monophosphate
NFkB: nuclear factor kappa B
PDE: phosphodiesterase
PKA: protein kinase A
SARS: severe acute respiratory syndrome
TNF alpha: tumor necrosis factor alpha
VCAM1: vascular cell adhesion molecule 1

## 1 INTRODUCTION

COVID‐19 is a disease caused by the SARS‐CoV‐2 virus, characterized by an early mild to moderate viral syndrome of fever, tiredness, cough, and headache.^1^ Over 80% of patients have a self‐limiting illness not requiring hospital admission and show clear improvement in two weeks. A minority of COVID‐19 patients progress through a transition phase around days 7‐11 of worsening pulmonary complications.^1^ These manifest as breathlessness, acute lung injury and respiratory failure, and often progress to require mechanical ventilation with subsequent high mortality. This deterioration appears to be driven by lung host responses including a cytokine storm of inflammation leading to severe tissue damage and irreversible organ failure likened to acute respiratory distress syndrome (ARDS).^2^ Patients who develop ARDS are at very high risk of death.

The cytokine storm phase of COVID‐19 is associated with increased production of a range of inflammatory cytokines including interleukin‐1 gamma (IL gamma), interleukin‐6 (IL6), tumor necrosis factor alpha (TNF alpha) and interferon gamma (IFN gamma).^3^ Several case series have reported increased TNF alpha levels in patients with COVID‐19 and particularly high levels appear to be associated with a severe disease course.^3^, ^4^ One series has described increased TNF alpha inducibility in macrophages, in the presence of SARS‐CoV‐2 virus.^4^ TNF alpha, as the master regulator of cytokines, is considered key in both immune pneumonitis and acute myocardial injury witnessed in COVID‐19.

Current COVID‐19 therapeutic studies are mainly focused on agents designed to target viral processes or virus‐host interactions, for example with Remdesivir. We note that Remdesivir has recently been approved by the UK government, after “numerical reduction in time to clinical improvement,” but failing to meet statistical significance on the primary clinical endpoint of mortality.^5^

An alternative therapeutic approach is to target the host responses that underlie the cytokine storm and associated inflammation.^3^ We, and other colleagues, have called for randomized clinical trials of anti‐TNF agents, such as Infliximab, to treat the cytokine storm induced by SARS‐CoV‐2.^6^, ^7^

### 1.1 Hypothesis

We hypothesize that pentoxifylline is an inexpensive anti‐TNF immuno‐modulator that will be effective in countering the cytokine storm in COVID‐19.

Pentoxifylline is a cytokine‐modulating anti‐inflammatory agent with many actions that might reasonably be expected to be therapeutic in the transition phase of COVID‐19. We first described the potential benefits of pentoxifylline as an inexpensive anti‐TNF for COVID‐19 in April 2020.^8^

### 1.2 Molecular and cellular actions of pentoxifylline

Pentoxifylline is a xanthine derivative drug with a wide range of actions at the cellular and molecular level. Pentoxifylline has rheological actions increasing erythrocyte deformability and was originally licensed for the treatment of peripheral vascular disease on the basis of suggested improvement of microvascular and capillary blood flow.^9^ More recently pentoxifylline has been determined to have extensive anti‐inflammatory properties.^10^ Pentoxifylline inhibits 5′‐nucleotidase and phosphodiesterase (PDE). PDE inhibition results in increased cAMP levels, increased protein kinase A (PKA) activity and altered transcriptional regulation of pro‐inflammatory genes through modulation of the NFkB/IkB pathway.^10^ Pentoxifylline also has beneficial therapeutic properties mediated by Adenosine A2A Receptor pathways in immune cells and in particular lung macrophages. Pentoxifylline downregulates transcription and expression levels of TNF alpha, IL1b, IL6, IFN gamma, ICAM1, and VCAM1. The 5′‐nucleotidase inhibition effect of pentoxifylline reduces the production of adenosine and inosine from adenosine monophosphate (AMP) and inosine monophosphate (IMP), respectively. Pentoxifylline appears to be able to downregulate the pathologically important pro‐inflammatory adenosine receptor A2A pathway (A2AR).^11^ These effects contribute to the extensive actions of pentoxifylline in reducing pro‐inflammatory signals. For example, pentoxifylline reduces cytokine release from pulmonary macrophages derived from patients with sarcoidosis.^12^ The lungs have the highest proportion of total resident macrophages in the human body at around 1 trillion.

### 1.3 Pentoxifylline therapy in pre‐clinical studies

In rats pentoxifylline downregulates a range of inflammatory cytokines in the context of sepsis and improves lung function.^12^ In animal studies pentoxifylline is effective as a therapeutic in a range of models of lung injury including radiation‐induced damage, cytotoxic agent damage and aortic clamping.^13^ The actions of pentoxifylline in reducing lung damage appear to be mediated by its effect on the adenosine receptor A2AR pathways.^11^ The direct inhibition of 5′‐nucleotidase activity by pentoxifylline likely reduces adenosine production and may therefore complement the actions on cytokine gene transcription. Pentoxifylline improves glomerular damage and reduces TNF alpha in crescentic glomerulonephritis in rats.^14^ Pentoxifylline does not appear to alter the replication of SARS‐CoV in mice and does not show direct antiviral activity.

### 1.4 Clinical studies of pentoxifylline

The anti‐inflammatory properties of pentoxifylline have been confirmed in a range of clinical studies in diverse organ failure syndromes. In severe renal disease patients exhibit resistance to erythropoietin through a pro‐inflammatory state characterized by increased TNF alpha, IFN gamma and IL6. Pentoxifylline improves erythropoietin sensitivity in these patients and this action is associated with reductions in serum TNF alpha and IFN gamma.^14^ In diabetic nephropathy pentoxifylline reduces proteinuria through inhibition of intraglomerular inflammatory signals. Clinical studies have demonstrated pentoxifylline as effective in reducing lung damage in the context of radiation damage, and cardiopulmonary bypass.^15^, ^16^ Ustunsoy et al (2003) for example found that pentoxifylline reduced levels of TNF alpha and IL6 when given at the time of cardiac bypass.^16^

One of the largest scale clinical trials of pentoxifylline anti‐inflammatory effects within the last decade, was the STOPAH trial.^17^ Examining potential benefit in acute alcoholic hepatitis, no survival benefit was demonstrated, however, no safety issues were reported in an inherently vulnerable, immunocompromised patient cohort. Pentoxifylline's preferential inhibition of macrophage function in alveoli, as opposed to hepatocytes, potentially explains this outcome.^18^

Rainsford (2006) reviewed possible treatments for the lung complications associated with inflammatory cytokines in H5N1 “bird flu” and suggested that pentoxifylline should be considered for clinical trials in view of its pharmacology and safety profile.^19^ These arguments appear equally suited to the case of COVID‐19. Although we cannot describe the SARS‐CoV‐2 pulmonary syndrome as identical to that seen in H5N1, there are certainly parallels.

TNF alpha has a pivotal role in orchestrating the production of a pro‐inflammatory cytokine cascade. TNF alpha is thus considered to be a “master regulator” of pro‐inflammatory cytokine production.^20^ Post‐mortem lung biopsies in COVID‐19 showed interstitial edema^21^ which would normally be the result of TNF induced increased capillary permeability.^22^ This non cardiogenic pulmonary edema (both interstitial and intra‐alveolar) is often the first stage of COVID‐19 acute lung injury that progresses through the cytokine storm to ARDS.^21^ The recent description of thrombosis and endothelialitis in COVID‐19^23^ raises the possibility that the rheological actions of pentoxifylline could be of benefit in maintaining microvascular function.

Pentoxifylline has over 50 years safety record data of use in humans and has an extensive evidence base for tolerability and safety. Nevertheless, its safety in the context of COVID‐19 has not been established and this would need close monitoring in a clinical trial setting. It is available in oral form with good bioavailability, and also can be delivered by intravenous injection. The usual dose orally is 1.2 g daily in three divided doses. An inhalational formulation has been developed, originally for use in neonates. In COVID‐19 the cytokine storm appears to be strongly centered in lung tissue, and accordingly inhaled pentoxifylline could be an optimum method for delivery at the highest concentrations where it is most effective, with minimal systemic exposure.

Use of a repurposed drug for COVID‐19 may have multiple advantages in addition to its safety and tolerability experience. It is widely available as a generic agent, with multiple sources of supply and therefore manageable cost. There should be no patent protection issues in redirecting the agent to trials in COVID‐19. This should be of acute interest as limited‐resource countries have now begun to amass cases of COVID‐19.

### 1.5 Design of studies of pentoxifylline in COVID‐19

A study of pentoxifylline in COVID‐19 should be feasible and ethical given the well‐described adverse event profile of the drug. A randomized study in COVID‐19 patients presenting with, or at high risk of, pulmonary complications could be designed with pentoxifylline versus a placebo or comparator treatment, alongside standard care. Pentoxifylline has been used at 400‐1200 mg daily in 400 mg doses. The initial study likely should use 1200 mg daily in divided doses. If there is initial evidence of benefit with oral pentoxifylline, then study of inhaled pentoxifylline could be valuable in selected patients. In the future it will also be logical to study the combination of cytokine‐modifying therapy with direct anti‐viral therapeutics (such as the recently favored Remdesivir). The efficacy of pentoxifylline can be assessed by randomized controlled trials with key endpoints including mortality, need for ventilatory support, time on ventilatory support, measures of oxygen exchange efficiency and time in hospital.

## 2 DISCUSSION

The full cellular, pre‐clinical and clinical profile of pentoxifylline suggests that pentoxifylline could be effective in reducing the severity of lung injury in patients with COVID‐19 primarily by its effect on TNF alpha. These actions could reduce the need for critical care interventions and reduce the burden and mortality of COVID‐19 in selected individuals.

Other colleagues have put forward hypotheses suggesting that the properties of pentoxifylline in relation to: (a) renin‐angiotensin‐aldosterone (RAS), (b) platelets and related coagulopathy and (c) rheological effects where there are microcirculatory disturbances, could all be important factors for the beneficial use of pentoxifylline as a therapeutic agent for COVID‐19. While these theories do have some basis, they have not yet been validated. Our central thesis, however, is that the primary benefit of pentoxifylline in COVID‐19 is on TNF alpha reduction. This effect has been shown to be clear cut in several clinical studies of other conditions similar to COVID‐19 as described above. We believe that TNF alpha is the central regulator of inflammation in COVID‐19 and, as such, targeting TNF alpha synthesis would be the most significant purpose for using pentoxifylline.

Closely following our comment to the Editor of the BMJ becoming available online,^8^ Maldonado et al put forward a similar hypothesis. However, in their article they ascribe the effect of pentoxifylline on the RAS system as an important potentially beneficial effect of the drug in COVID‐19. Their contention was based on the observed effect of pentoxifylline on Type 1 Angiotensin II receptors (AT1) in patients with hyper activation of RAS.^24^

RAS hyper‐activation has been speculated upon, as a cause of worsening lung injury in COVID‐19 patients, based on the fact that SARS‐CoV‐2 spike protein binds to Angiotensin Converting Enzyme (ACE) 2 receptors in pulmonary alveolar cells. However, these assumptions have not been validated in observational studies. Studies have not shown any evidence of greater disease severity in patients already on ACE inhibitors who developed COVID‐19, which would have been expected if these assumptions were valid.^25^

The beneficial effects of pentoxifylline on lung injury are mediated by G protein coupled Adenosine A2 receptors.^26^ Adenosine has multiple physiological effects through membrane‐bound receptors linked to G proteins. There are four subtypes of adenosine receptors: A_1_AR, A_2A_AR, A_2B_AR, and A_3_AR. Pentoxifylline has an antagonist effect on adenosine binding with its receptor (A2aAR) and functions as an anti‐inflammatory agent.^27^

A1AR is predominantly found in the central nervous system and kidneys and fewer receptors are found in lungs. Pentoxifylline has not been shown to have any effect on A1AR.^26^ A2a Adenosine Receptors are mainly found in lungs and immune cells and the pentoxifylline beneficial effect is mediated by the A2aAR pathway.^27^ A2bAR has an antagonistic effect when stimulated. However, the affinity is low and there is no evidence to support the binding of pentoxifylline to A2bAR. A3AR is also unaffected by pentoxifylline.^27^

Activation of NLRP3 Inflammasome protein by SARS‐CoV‐2 viral infection in the early stages has been put forward as a hypothesis by Ratajczak and Lucia.^28^ The predominant effect of pentoxifylline via A2AR pathways on both lung macrophages and immune cells is anti‐inflammatory, with reduced TNF alpha, Interleukin‐12, Interleukin‐6, Interleukin‐8 levels and increased Interleukin‐10 levels. The balance between the anti‐cytokine A2AR mediated action of pentoxifylline and any theoretical gradient dependent passage of adenosine back into the cell to re‐activate NLRP3 inflammasome protein appears to clearly favor the A2AR effect of pentoxifylline in terms of cytokine outcomes.

Although pentoxifylline has rheological effects which is the main indication for its long‐standing use in peripheral vascular disease, no clinical benefit has been reported from controlled trials in coronary artery disease or cerebrovascular disease. Although pulmonary microcirculatory abnormalities were found on post‐mortem studies in COVID‐19,^21^, ^23^ pentoxifylline is unlikely to confer any therapeutic benefits in the pulmonary circulation because its rheological benefit has only been shown to be of clinical benefit in peripheral limb vessels.

In a significant minority of COVID‐19 patients, coagulopathy has been found in severe COVID‐19 with detection of raised D‐dimers.^29^ However, no clinical trial has shown any benefit with anti‐platelet therapy and the minor in‐vitro platelet inhibition associated with pentoxifylline may not confer any therapeutic benefit related to this, in COVID‐19.^29^

If the theoretical therapeutic benefit of pentoxifylline for COVID‐19 based on its anti‐Tumor Necrosis Factor effect is clinically demonstrable, it may prove to be an inexpensive, and readily available, treatment strategy to target harmful cytokine excess in this disease.

We advocate urgent randomized trials of pentoxifylline for patients infected with SARS CoV‐2.

## 3 DECLARATION

BMH has received honoraria for speaking or consulting from AstraZeneca, Gilead Sciences, Otsuka, Retrophin, Sanofi, UCB and ViiV Healthcare.

## CONFLICT OF INTEREST

None of the authors have any relevant actual or potential conflicts of interest.

## AUTHOR CONTRIBUTION

All authors contributed equally.
